# The Validity of Video Fall Detection for Assisted Living Residents with Dementia

**DOI:** 10.1093/geroni/igaf122.2644

**Published:** 2025-12-31

**Authors:** London Jones, Scott Davis, Philip Sloane, Elizabeth Peiffer, Shirley Nickels, Sheryl Zimmerman

**Affiliations:** The University of North Carolina at Chapel Hill, Chapel Hill, North Carolina, United States; University of North Carolina at Chapel Hill, Chapel Hill, North Carolina, United States; University of North Carolina at Chapel Hill, Chapel Hill, North Carolina, United States; SafelyYou, San Francisco, California, United States; SafelyYou, San Francisco, California, United States; University of North Carolina at Chapel Hill, Chapel Hill, North Carolina, United States

## Abstract

Roughly 25% of older adults fall each year, with rates almost twice as high among persons with dementia. Reducing falls in assisted living (AL) is especially important, given that AL is the residential setting housing most people with dementia. Unfortunately, many falls are unwitnessed and under reported, making it difficult to respond promptly and effectively. Video monitoring has emerged as a promising method of detecting falls, but the extent to which it can be used for research has not been evaluated. This study examined the validity of identifying a fall using recorded video events compared to an observational gold standard. A team of four researchers were trained to categorize falls using prescribed criteria, and achieved 100% accuracy. Then, they independently reviewed 100 video clips (50 ‘fall’ and 50 ‘non-fall’) from a video database of “residents on the ground” and compared the ratings to the gold standard. Residents from six different AL communities and no more than four videos of the same resident were included. Overall sensitivity was 82%; raters correctly rated 41 of 50 videos that contained falls. Specificity was 93.2%; 41 of 44 videos in which the raters identified a fall met the gold standard definition of a fall. When the initiation of descent was considered, perfect agreement was achieved for descent from standing, squatting or kneeling. Challenges arose when individuals slowly descended from a bed to the floor. Based on these results, video capture of falls is a valid data source to examine, and potentially prevent, falls.

